# Crystal structure of (η^4^-cyclo­octa­diene)(3,3′-dimesityl-1,1′-methyl­enediimidazoline-2,2′-diyl­idene)nickel(0) tetra­hydro­furan monosolvate

**DOI:** 10.1107/S2056989018012252

**Published:** 2018-09-07

**Authors:** Carlos D. Yamamoto, Zijie Zhang, Sabine Chantal E. Stieber

**Affiliations:** a Department of Chemistry & Biochemistry, California State Polytechnic University, Pomona, 3801 W. Temple Ave., Pomona, CA 91768, USA

**Keywords:** crystal structure, inorganic chemistry, organometallic chemistry, NHC, nickel, carbene

## Abstract

The crystal structure of the title compound, [Ni(C_25_H_28_N_4_)(C_8_H_12_)]·C_4_H_8_O or (^Mes^NHC_2_Me)Ni(COD), which contains a bidentate N-heterocyclic carbene (NHC) ligand with mesityl aryl groups is reported. The complex at 100 K has monoclinic (*P*2_1_/*c*) symmetry and a distorted tetra­hedral geometry around the nickel center, with the cyclo­octa­diene ligand coordinated in a κ^2^,η^2^ fashion. The bidentate NHC ligand is not planar, with a C(carbene)—Ni—C(carbene) angle of 91.51 (12)°, resulting in the mesityl groups being on the same side of the cyclo­octa­diene (COD) ligand. One mol­ecule of tetra­hydro­furan (THF) is co-crystallized with the nickel complex and has positional disorder.

## Abstract 2

The complex at 100 K has monoclinic (*P*2_1_/*c*) symmetry and a distorted tetra­hedral geometry around the nickel center, with the cyclo­octa­diene ligand coordinated in a κ^2^,η^2^ fashion. The bidentate NHC ligand is not planar, with a C(carbene)—Ni—C(carbene) angle of 91.51 (12)°, resulting in the mesityl groups being on the same side of the cyclo­octa­diene (COD) ligand.

## Chemical context   

N-heterocyclic carbene (NHC) ligands, which have found extensive use in catalysis and organometallic chemistry, coordinate to metal centers *via* the lone pair of electrons of the carbene (Arduengo, 1999 ▸; Hopkinson *et al.*, 2014 ▸; Lummiss *et al.*, 2015 ▸). Bidentate NHC ligands (NHC_2_) may be formed by linking two NHC ligands together; however, coordination to first row transition metals has been limited (Brendel *et al.*, 2014 ▸; Herrmann *et al.*, 1999 ▸; Douthwaite *et al.*, 1999 ▸; Huffer *et al.*, 2013 ▸; Harrold & Hillhouse, 2013 ▸). Nickel(0)cyclo­octa­diene complexes with {1,1′-di(isoprop­yl)phenyl-3,3′-methyl­ene­di­imid­azolin-2,2′-diyl­idene} and {1,1′-tert(but­yl)-3,3′-methyl­ene­diimidazolin-2,2′-diyl­idene} ligands have been reported, but the mesityl variant is not known (Brendel *et al.*, 2014 ▸). Herein, a synthetic procedure for the synthesis of {1,1′-di(mes­it­yl)-3,3′-methyl­enediimidazolin-2,2′-diyl­idene}nickel(0)cyclo­octa­diene, (^Mes^NHC_2_Me)Ni(COD), and its crystallographic characterization are reported.
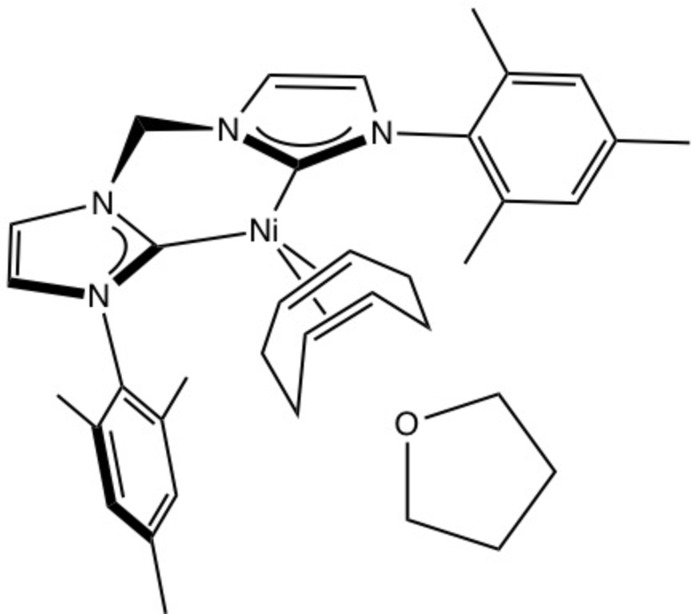


## Structural commentary   

**Figure 1:**
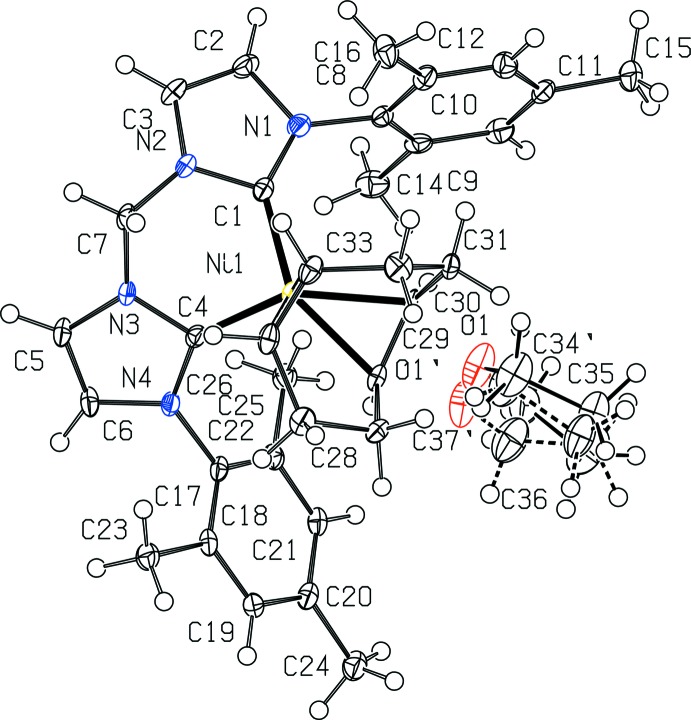
View of (^Mes^NHC_2_Me)Ni(COD)·THF with 50% probability ellipsoids, showing the THF disorder.

**Figure 2:**
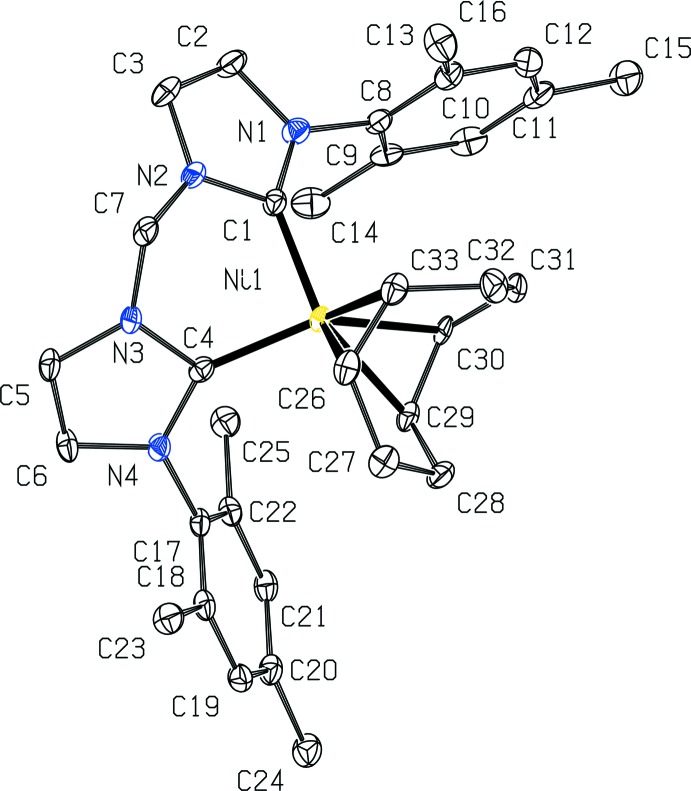
View of one mol­ecule of (^Mes^NHC_2_Me)Ni(COD) with 50% probability ellipsoids. The THF mol­ecules and H atoms are omitted for clarity.

(^Mes^NHC_2_Me)Ni(COD) co-crystallizes with one mol­ecule of tetra­hydro­furan (THF) as shown in Fig. 1 ▸. Fig. 2 ▸ depicts the structure without the THF for clarity. The nickel(0) center has a pseudo-tetra­hedral geometry, being coordinated to (^Mes^NHC_2_Me) in a κ^2^ fashion with a C1—Ni1—C4 angle of 91.51 (12)° and to COD in a κ^2^,η^2^ fashion. The distances between the nickel center and the (^Mes^NHC_2_Me) ligand are 1.909 (3) Å for Ni1—C1 and 1.916 (3) Å for Ni1—C4. These are slightly shorter than the analagous distances of 1.938 (3) and 1.953 (3) Å, respectively, reported for (^Dipp^NHC_2_Me)Ni(COD) (Brendel *et al.*, 2014 ▸). The distances from the nickel center to the COD ligand are 1.921 (3) and 2.018 (3) Å as measured from Ni1 to the mid-points of C29—C30 and C26—C33, respectively. The backbone of each NHC contains unsaturated C=C double bonds, as evidenced by bond distances of 1.344 (4) Å for C2—C3 and 1.341 (4) Å for C5—C6. The other NHC backbone distances are 1.390 (4) Å for N1—C2, 1.384 (4) Å for N2—C3, 1.388 (3) Å for N3—C5, and 1.395 (3) Å for N4—C6. The remaining NHC bond lengths to the carbene are 1.377 (4) Å for N1—C1, 1.374 (3) Å for N2—C1, 1.379 (3) Å for N3—C4, and 1.374 (3) Å for N4—C4. These are comparable to the analagous NHC carbene distances reported for (^Dipp^NHC_2_Me)Ni(COD) of 1.374 (4), 1.387 (4), 1.379 (4), and 1.386 (4) Å, respectively (Brendel *et al.*, 2014 ▸). The portions of the COD ligand that are coordin­ated to nickel have C=C bond distances of 1.411 (4) Å for C29=C30 and 1.374 (4) Å for C26=C33, consistent with unsaturated double bonds. These are slightly longer than the analagous C=C COD distances reported for (^Dipp^NHC_2_Me)Ni(COD) of 1.383 (5), and 1.355 (5) Å, respectively (Brendel *et al.*, 2014 ▸). The remaining C—C bond distances of the COD fragment are in the range of 1.512 (4)–1.539 (4) Å, consistent with saturated C—C single bonds, and comparable to the range of bond lengths reported for (^Dipp^NHC_2_Me)Ni(COD) of 1.502–1.529 Å (Brendel *et al.*, 2014 ▸).

## Supra­molecular features   

**Figure 3:**
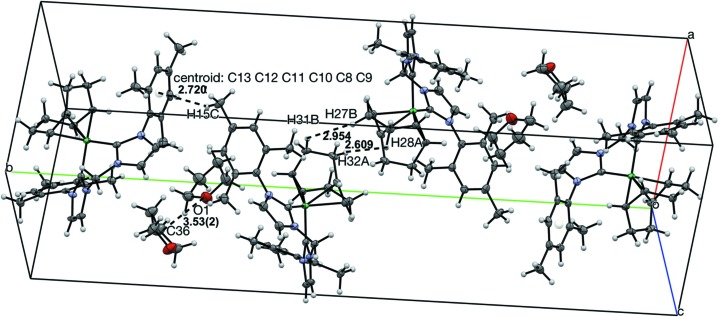
View of four mol­ecules of (^Mes^NHC_2_Me)Ni(COD) and THF in the unit cell with 50% probability ellipsoids, highlighting inter­molecular distances. Distances between H atoms are listed without standard deviations because the H atoms were positionally fixed.

Four mol­ecules of (^Mes^NHC_2_Me)Ni(COD) and THF are present in the unit cell, as depicted in Fig. 3 ▸. The mol­ecules are oriented such that the COD ligands from neighboring mol­ecules are adjacent to each other, with distances of 2.61 and 2.95 Å between nearest hydrogen atoms (H28*A*⋯H32*A* and H27*B*⋯H31*B*, respectively). Standard deviations for distances including hydrogen atoms are not listed because hydrogen atoms were positionally fixed. The methyl group at the *para* position of the mesityl fragment is oriented towards the aryl ring of the mesityl of the neighboring mol­ecule, with a distance of 2.72 Å between the aryl ring centroid (C8–C13) and the nearest methyl hydrogen atom (H15*C*). The THF mol­ecule is closest to the backbone of the (^Mes^NHC_2_Me) ligand, such that the mol­ecules are 3.527 (17) Å apart from one oxygen atom (O1) to the next nearest carbon atom (C36) (Table 1 ▸).

## Database survey   

A survey of the Cambridge Structural Database (Web accessed August 9, 2018; Groom *et al.*, 2016 ▸) and SciFinder (SciFinder, 2018 ▸) yielded no exact matches for this complex, but related complexes with slightly varied ligands, such as (^tBu^NHC_2_Me)Ni(COD) (tBu = *tert*-but­yl) and (^Dipp^NHC_2_Me)Ni(COD) (Dipp = 2,6-di(isoprop­yl)phen­yl) (Brendel *et al.*, 2014 ▸) have been reported. The crystal structures of both these complexes have generally similar structural characteristics. The main difference is that the COD ligand in (^tBu^NHC_2_Me)Ni(COD) is coordinated in a κ^1^,η^2^ fashion.

## Synthesis and crystallization   

1-(2,4,6-Tri­methyl­phen­yl)-1*H*-imidazole, and 1,1′-di(mesit­yl)-3,3′-methyl­ene-diimidazolium dibromide were synthesized according to literature procedures (Liu *et al.*, 2003 ▸; Gardiner *et al.*, 1999 ▸). 1,1′-Di(mesit­yl)-3,3′-methyl­ene-diimidazolium dibromide was dried overnight on a high vacuum line before transferring to an inert atmosphere N_2_ glovebox. {1,1′-Di(mesit­yl)-3,3′-methyl­enediimidazolin-2,2′-diyl­idene}nickel(0)cyclo­octa­diene was synthesized by the following method. A 20 mL scintillation vial was charged with 0.203 g (0.366 mmol, 1 eq.) of 1,1′-di(mesit­yl)-3,3′-methyl­ene-diimidazolium dibromide, approximately 10 mL of tetra­hydro­furan and a stirbar. 1.80 mL (0.915 mmol, 2.5 eq.) of 0.5 *M* potassium bis­(tri­methyl­sil­yl)amide in toluene were added dropwise to the solution while stirring, resulting in a color change to blue–green. The mixture was stirred for approximately five h, resulting in a clear orange–brown solution, which was filtered through a glass frit with celite. The filtrate was transferred to a new 20 mL glass scintillation vial and stirred while adding 0.090 g (0.329 mmol, 0.9 eq.) of bis­(1,5-cyclo­octa­diene)nickel(0). The mixture was stirred for 4–12 h, resulting in a clear dark red–orange solution. The solvent was removed *in vacuo*, and the orange solid was washed with pentane (3–5 washes of approximately 10 mL), resulting in 0.151 g (78%) of an orange solid identified as {1,1′-di(mesit­yl)-3,3′-methyl­enediimidazolin-2,2′-diyl­idene}nickel(0)cyclo­octa­diene. Single crystals suitable for X-ray analysis were grown from a dilute solution of pentane with a drop of tetra­hydro­furan. ^1^H NMR (399.777 MHz, C_6_D_6_, 295 K): δ = 1.96–2.12 (*m*, 8H; C*H*_2_-COD), 2.13 (*s*, 6H; C*H*_3_ *p*-mesit­yl), 2.17 (*s*, 12H; C*H*_3_ *o*-mesit­yl), 4.07 (*s*, 4H; C*H*-Ni-COD), 4.68 (*s*, 2H, C*H*_2_), 6.12 (*s*, 2H, C*H*-Im), 6.42 (*s*, 2H, C*H*-Im), 6.84 (*s*, 4H, *m*-C*H*-*Ar*). ^13^C NMR (101 MHz, C_6_D_6_, 295 K): δ = 18.43 (*C*H_3_ *o*-mesit­yl), 21.14 (*C*H_3_ *p*-mesit­yl), 32.51 (*C*H_2_-COD), 61.31 (*C*H_2_), 74.17 (*C*H-Ni-COD), 118.18 (*C*H-Im), 119.81 (*C*H-Im), 128.94 (*m*-*C*H-*Ar*), 136.22 (*o*-*C*-*Ar*), 137.89 (*p*-*C*-*Ar*), 138.91 (*i*-*C*-*Ar*), 205.37 (N_2_ *C*-Im).

## Refinement   

Crystal data, data collection and structure refinement details are summarized in Table 2 ▸. Most hydrogen atoms were placed in calculated positions using the AFIX commands of *SHELXL* and refined as riding with distances of 0.95 Å for C—H, 0.99 Å for CH_2_ and 0.98 Å for CH_3_. Methyl H atoms were allowed to rotate but not to tip to best fit the experimental electron density. *U*_iso_ values of riding H atoms were set to 1.2 times *U*_eq_(C) for CH and CH_2_, and 1.5 times *U*_eq_(C) for CH_3_. The positions of the hydrogen atoms on the portions of the COD ligand directly bound to nickel and attached to C26, C29, C30, and C33 were determined from the difference map. Positions and isotropic displacement parameters were refined, but the associated C—H atom distances were restrained to be similar to each other by using a SADI command of *SHELXL* (for C26—H26*A*, C29—H29*A*, C30—H30*A*, and C33—H33*A*).

The two moieties of the disordered THF mol­ecule were restrained to have similar geometries (a SAME command in *SHELXL* was applied for O1′ through C37′ and O1 through C34 to make bond distances and angles equivalent with standard deviations of 0.02 and 0.04 Å for 1,2- and 1,3 distances, respectively). *U* ^ij^ components of ADPs of the disordered atoms were restrained to be similar to each other with an esd of 0.01 Å^2^ for atoms closer to each other than 2.0 Å (SIMU command of *SHELXL*), resulting in a final close-to-equal site occupancy ratio of 0.502 (13) to 0.498 (13).

## Supplementary Material

Crystal structure: contains datablock(s) I. DOI: 10.1107/S2056989018012252/zl2737sup1.cif

Structure factors: contains datablock(s) I. DOI: 10.1107/S2056989018012252/zl2737Isup2.hkl

CCDC reference: 1864396

Additional supporting information:  crystallographic information; 3D view; checkCIF report

## Figures and Tables

**Table 1:** Inter­molecular distances in the unit cell of (MesNHC_2_Me)Ni(COD) Standard deviations for distances including H atoms are omitted because H atoms were positionally fixed.

	Distance (Å)
H15*C*⋯centroid(C8–C13)	2.72
H27*B*⋯H31*B*	2.95
H28*A*⋯H32*A*	2.61
O1⋯C36	3.527 (17)

**Table 2:** Experimental details

Crystal data	
Chemical formula	[Ni(C_25_H_28_N_4_)(C_8_H_12_)]·C_4_H_8_O
*M*_r_	623.50
Crystal system, space group	Monoclinic, *P*2_1_/*c*
Temperature (K)	100
*a*, *b*, *c* (Å)	10.5557 (7), 35.308 (2), 8.5951 (5)
β (°)	99.591 (2)
*V* (Å^3^)	3158.6 (3)
*Z*	4
Radiation type	Mo *K*α
μ (mm^−1^)	0.65
Crystal size (mm)	0.53 × 0.15 × 0.04

Data collection	
Diffractometer	Bruker D8 Venture Kappa
Absorption correction	Multi-scan (*SADABS*; Krause *et al.*, 2015 ▸)
*T*_min_, *T*_max_	0.658, 0.746
No. of measured, independent and observed [*I* > 2σ(*I*)] reflections	61849, 6973, 4784
*R*_int_	0.141
(sin θ/λ)_max_ (Å^−1^)	0.642

Refinement	
*R*[*F* ^2^ > 2σ(*F* ^2^)], *wR*(*F* ^2^), *S*	0.048, 0.104, 1.03
No. of reflections	6973
No. of parameters	456
No. of restraints	182
H-atom treatment	H atoms treated by a mixture of independent and constrained refinement
Δρ_max_, Δρ_min_ (e Å^−3^)	0.50, −0.46

Computer programs: *APEX3* and *SAINT* (Bruker, 2017 ▸), *SHELXT2014* (Sheldrick, 2015*a* ▸), *SHELXL2016* (Sheldrick, 2015*b* ▸), *Mercury* (Macrae *et al.*, 2006 ▸) and *publCIF* (Westrip, 2010 ▸).

## supplementary crystallographic information

### Crystal data

**Table:**

[Ni(C_25_H_28_N_4_)(C_8_H_12_)]·C_4_H_8_O	*F*(000) = 1336
*M**_r_* = 623.50	*D*_x_ = 1.311 Mg m^−^^3^
Monoclinic, *P*2_1_/*c*	Mo *K*α radiation, λ = 0.71073 Å
*a* = 10.5557 (7) Å	Cell parameters from 5806 reflections
*b* = 35.308 (2) Å	θ = 4.6–54.2°
*c* = 8.5951 (5) Å	µ = 0.65 mm^−^^1^
β = 99.591 (2)°	*T* = 100 K
*V* = 3158.6 (3) Å^3^	Plate, orange
*Z* = 4	0.53 × 0.15 × 0.04 mm

### Data collection

**Table:**

Bruker D8 Venture Kappa diffractometer	4784 reflections with *I* > 2σ(*I*)
Radiation source: microfocus sealed tube	*R*_int_ = 0.141
φ and ω scans	θ_max_ = 27.2°, θ_min_ = 2.6°
Absorption correction: multi-scan (SADABS; Krause *et al.*, 2015)	*h* = −13→13
*T*_min_ = 0.658, *T*_max_ = 0.746	*k* = −45→45
61849 measured reflections	*l* = −10→11
6973 independent reflections	

### Refinement

**Table:**

Refinement on *F*^2^	Primary atom site location: structure-invariant direct methods
Least-squares matrix: full	Secondary atom site location: difference Fourier map
*R*[*F*^2^ > 2σ(*F*^2^)] = 0.048	Hydrogen site location: mixed
*wR*(*F*^2^) = 0.104	H atoms treated by a mixture of independent and constrained refinement
*S* = 1.03	*w* = 1/[σ^2^(*F*_o_^2^) + (0.0168*P*)^2^ + 5.0058*P*] where *P* = (*F*_o_^2^ + 2*F*_c_^2^)/3
6973 reflections	(Δ/σ)_max_ < 0.001
456 parameters	Δρ_max_ = 0.50 e Å^−^^3^
182 restraints	Δρ_min_ = −0.46 e Å^−^^3^

### Special details

**Table:**

Geometry. All esds (except the esd in the dihedral angle between two l.s. planes) are estimated using the full covariance matrix. The cell esds are taken into account individually in the estimation of esds in distances, angles and torsion angles; correlations between esds in cell parameters are only used when they are defined by crystal symmetry. An approximate (isotropic) treatment of cell esds is used for estimating esds involving l.s. planes.

### Fractional atomic coordinates and isotropic or equivalent isotropic displacement parameters (Å^2^)

**Table:**

	*x*	*y*	*z*	*U*_iso_*/*U*_eq_	Occ. (<1)
Ni01	0.40659 (3)	0.59095 (2)	0.82589 (4)	0.01352 (10)	
N1	0.5497 (2)	0.66097 (7)	0.9799 (3)	0.0171 (5)	
N2	0.4717 (2)	0.62336 (7)	1.1329 (3)	0.0160 (5)	
N3	0.2805 (2)	0.58667 (7)	1.0943 (3)	0.0158 (5)	
N4	0.1305 (2)	0.58132 (7)	0.8953 (3)	0.0155 (5)	
C1	0.4848 (3)	0.62709 (8)	0.9772 (3)	0.0147 (6)	
C2	0.5758 (3)	0.67656 (9)	1.1304 (3)	0.0217 (7)	
H2	0.620362	0.699526	1.159441	0.026*	
C3	0.5259 (3)	0.65294 (9)	1.2269 (3)	0.0209 (7)	
H3	0.527500	0.655931	1.337005	0.025*	
C4	0.2613 (3)	0.58607 (8)	0.9316 (3)	0.0144 (6)	
C5	0.1676 (3)	0.58212 (8)	1.1549 (3)	0.0179 (6)	
H5	0.158714	0.581583	1.263073	0.021*	
C6	0.0735 (3)	0.57865 (8)	1.0301 (3)	0.0184 (6)	
H6	−0.015291	0.575044	1.032910	0.022*	
C7	0.4080 (3)	0.59040 (9)	1.1849 (3)	0.0165 (6)	
H7A	0.458691	0.567380	1.171654	0.020*	
H7B	0.402361	0.592985	1.298263	0.020*	
C8	0.5968 (3)	0.67746 (8)	0.8478 (3)	0.0163 (6)	
C9	0.5204 (3)	0.70228 (8)	0.7464 (3)	0.0177 (6)	
C10	0.5734 (3)	0.71794 (8)	0.6224 (4)	0.0214 (7)	
H10	0.521938	0.734348	0.550278	0.026*	
C11	0.6975 (3)	0.71046 (8)	0.6009 (3)	0.0201 (7)	
C12	0.7709 (3)	0.68572 (8)	0.7052 (3)	0.0200 (7)	
H12	0.856658	0.680319	0.691993	0.024*	
C13	0.7218 (3)	0.66878 (8)	0.8281 (3)	0.0178 (6)	
C14	0.3861 (3)	0.71230 (9)	0.7685 (4)	0.0262 (7)	
H14A	0.330746	0.714082	0.665288	0.039*	
H14B	0.387035	0.736703	0.823046	0.039*	
H14C	0.353120	0.692657	0.831690	0.039*	
C15	0.7554 (3)	0.72843 (10)	0.4696 (4)	0.0282 (8)	
H15A	0.795053	0.708790	0.413047	0.042*	
H15B	0.820795	0.746881	0.514294	0.042*	
H15C	0.687898	0.741271	0.396340	0.042*	
C16	0.8014 (3)	0.64084 (9)	0.9349 (4)	0.0253 (7)	
H16A	0.753695	0.617055	0.936258	0.038*	
H16B	0.820161	0.651211	1.042069	0.038*	
H16C	0.882059	0.636053	0.896079	0.038*	
C17	0.0553 (3)	0.58069 (8)	0.7392 (3)	0.0144 (6)	
C18	−0.0086 (3)	0.54770 (8)	0.6843 (3)	0.0158 (6)	
C19	−0.0857 (3)	0.54843 (8)	0.5365 (3)	0.0169 (6)	
H19	−0.129619	0.526032	0.497217	0.020*	
C20	−0.1002 (3)	0.58104 (8)	0.4447 (3)	0.0171 (6)	
C21	−0.0343 (3)	0.61332 (8)	0.5027 (3)	0.0171 (6)	
H21	−0.042410	0.635603	0.439830	0.021*	
C22	0.0438 (3)	0.61413 (8)	0.6504 (3)	0.0167 (6)	
C23	−0.0007 (3)	0.51193 (8)	0.7823 (3)	0.0210 (7)	
H23A	−0.016531	0.489902	0.712349	0.032*	
H23B	−0.065404	0.512797	0.851747	0.032*	
H23C	0.085074	0.509905	0.846178	0.032*	
C24	−0.1874 (3)	0.58143 (9)	0.2862 (3)	0.0229 (7)	
H24A	−0.147939	0.566754	0.210144	0.034*	
H24B	−0.200454	0.607603	0.249046	0.034*	
H24C	−0.270478	0.570174	0.296654	0.034*	
C25	0.1133 (3)	0.64962 (8)	0.7106 (3)	0.0201 (7)	
H25A	0.206066	0.644930	0.729324	0.030*	
H25B	0.086099	0.657291	0.809584	0.030*	
H25C	0.093035	0.669840	0.632288	0.030*	
C26	0.4432 (3)	0.53181 (8)	0.8337 (3)	0.0186 (7)	
H26A	0.423 (2)	0.5236 (7)	0.932 (2)	0.010 (7)*	
C27	0.3628 (3)	0.51489 (9)	0.6886 (3)	0.0208 (7)	
H27A	0.276896	0.508545	0.713097	0.025*	
H27B	0.403403	0.491004	0.661976	0.025*	
C28	0.3460 (3)	0.54110 (8)	0.5439 (3)	0.0192 (7)	
H28A	0.417278	0.536693	0.484457	0.023*	
H28B	0.264580	0.534738	0.473722	0.023*	
C29	0.3440 (3)	0.58277 (8)	0.5894 (3)	0.0163 (6)	
H29A	0.262 (2)	0.5947 (8)	0.559 (3)	0.014 (8)*	
C30	0.4559 (3)	0.60531 (8)	0.6134 (3)	0.0149 (6)	
H30A	0.443 (3)	0.6319 (6)	0.597 (4)	0.024 (9)*	
C31	0.5874 (3)	0.59020 (9)	0.5982 (3)	0.0189 (6)	
H31A	0.651953	0.610249	0.630734	0.023*	
H31B	0.588887	0.584307	0.485938	0.023*	
C32	0.6263 (3)	0.55448 (9)	0.6975 (4)	0.0230 (7)	
H32A	0.607565	0.531937	0.629023	0.028*	
H32B	0.720140	0.555157	0.735062	0.028*	
C33	0.5586 (3)	0.55016 (9)	0.8387 (3)	0.0192 (7)	
H33A	0.612 (2)	0.5536 (8)	0.940 (3)	0.014 (8)*	
O1	0.1468 (10)	0.7119 (3)	0.3805 (9)	0.0435 (18)	0.502 (13)
C34	0.2266 (17)	0.6845 (6)	0.3253 (16)	0.041 (2)	0.502 (13)
H34A	0.200127	0.658717	0.351848	0.049*	0.502 (13)
H34B	0.317128	0.688406	0.375222	0.049*	0.502 (13)
C35	0.2128 (11)	0.6890 (4)	0.1491 (15)	0.037 (2)	0.502 (13)
H35A	0.283351	0.704611	0.120317	0.045*	0.502 (13)
H35B	0.212307	0.664092	0.096250	0.045*	0.502 (13)
C36	0.0855 (17)	0.7087 (4)	0.1057 (14)	0.044 (2)	0.502 (13)
H36A	0.014923	0.690064	0.079960	0.052*	0.502 (13)
H36B	0.085608	0.725673	0.014167	0.052*	0.502 (13)
C37	0.0717 (10)	0.7310 (3)	0.2521 (10)	0.0352 (19)	0.502 (13)
H37A	0.103029	0.757211	0.244043	0.042*	0.502 (13)
H37B	−0.019410	0.731908	0.266236	0.042*	0.502 (13)
O1'	0.0905 (10)	0.7022 (2)	0.3664 (9)	0.0391 (17)	0.498 (13)
C34'	0.2015 (16)	0.6844 (6)	0.3267 (16)	0.041 (2)	0.498 (13)
H34C	0.213464	0.658950	0.375138	0.049*	0.498 (13)
H34D	0.279163	0.699780	0.363667	0.049*	0.498 (13)
C35'	0.1769 (12)	0.6816 (3)	0.1473 (15)	0.037 (2)	0.498 (13)
H35C	0.131992	0.657687	0.111634	0.045*	0.498 (13)
H35D	0.258105	0.682994	0.104300	0.045*	0.498 (13)
C36'	0.0919 (16)	0.7159 (4)	0.0979 (13)	0.037 (2)	0.498 (13)
H36C	0.143422	0.738695	0.084198	0.044*	0.498 (13)
H36D	0.030569	0.711026	−0.000385	0.044*	0.498 (13)
C37'	0.0240 (12)	0.7195 (3)	0.2392 (11)	0.046 (2)	0.498 (13)
H37C	0.013576	0.746664	0.263105	0.056*	0.498 (13)
H37D	−0.062699	0.708117	0.213787	0.056*	0.498 (13)

### Atomic displacement parameters (Å^2^)

**Table:**

	*U*^11^	*U*^22^	*U*^33^	*U*^12^	*U*^13^	*U*^23^
Ni01	0.01553 (18)	0.01667 (19)	0.00880 (17)	−0.00028 (16)	0.00334 (13)	−0.00042 (16)
N1	0.0191 (13)	0.0178 (14)	0.0137 (12)	−0.0008 (10)	0.0009 (10)	0.0001 (10)
N2	0.0189 (13)	0.0194 (14)	0.0095 (12)	−0.0007 (10)	0.0016 (10)	−0.0006 (10)
N3	0.0177 (12)	0.0209 (14)	0.0098 (11)	−0.0016 (11)	0.0050 (9)	0.0011 (10)
N4	0.0163 (12)	0.0202 (14)	0.0104 (12)	−0.0001 (10)	0.0033 (10)	0.0025 (10)
C1	0.0147 (14)	0.0183 (16)	0.0111 (14)	0.0021 (12)	0.0020 (11)	0.0004 (12)
C2	0.0287 (18)	0.0203 (17)	0.0153 (15)	−0.0043 (14)	0.0017 (13)	−0.0059 (12)
C3	0.0269 (17)	0.0241 (17)	0.0106 (15)	0.0016 (14)	−0.0001 (13)	−0.0035 (12)
C4	0.0180 (14)	0.0144 (15)	0.0105 (13)	−0.0003 (12)	0.0013 (11)	0.0012 (11)
C5	0.0197 (15)	0.0237 (17)	0.0116 (14)	0.0033 (13)	0.0067 (12)	0.0028 (12)
C6	0.0171 (15)	0.0243 (17)	0.0157 (15)	0.0035 (12)	0.0083 (12)	0.0057 (12)
C7	0.0218 (15)	0.0195 (15)	0.0082 (13)	0.0002 (13)	0.0022 (11)	0.0042 (12)
C8	0.0214 (15)	0.0154 (15)	0.0118 (14)	−0.0042 (12)	0.0020 (12)	−0.0018 (11)
C9	0.0206 (16)	0.0125 (15)	0.0184 (16)	−0.0035 (12)	−0.0018 (12)	−0.0029 (12)
C10	0.0284 (18)	0.0139 (16)	0.0189 (16)	−0.0011 (13)	−0.0046 (13)	0.0050 (12)
C11	0.0309 (18)	0.0171 (16)	0.0114 (15)	−0.0062 (13)	0.0010 (13)	−0.0008 (12)
C12	0.0214 (16)	0.0193 (17)	0.0196 (16)	−0.0003 (13)	0.0048 (13)	−0.0003 (13)
C13	0.0232 (16)	0.0157 (16)	0.0139 (15)	0.0001 (13)	0.0016 (12)	0.0004 (12)
C14	0.0250 (18)	0.0223 (18)	0.0292 (18)	0.0006 (14)	−0.0018 (14)	0.0002 (14)
C15	0.037 (2)	0.0299 (19)	0.0182 (17)	−0.0086 (16)	0.0055 (14)	0.0058 (14)
C16	0.0259 (17)	0.0259 (19)	0.0248 (18)	0.0037 (14)	0.0060 (14)	0.0118 (14)
C17	0.0130 (14)	0.0207 (16)	0.0104 (14)	0.0035 (12)	0.0041 (11)	0.0023 (11)
C18	0.0158 (14)	0.0192 (16)	0.0136 (15)	0.0020 (12)	0.0062 (12)	0.0045 (12)
C19	0.0165 (15)	0.0197 (16)	0.0151 (15)	−0.0016 (12)	0.0047 (12)	0.0017 (12)
C20	0.0159 (14)	0.0233 (17)	0.0124 (14)	0.0048 (12)	0.0032 (11)	0.0022 (12)
C21	0.0193 (15)	0.0189 (16)	0.0144 (15)	0.0030 (12)	0.0063 (12)	0.0048 (12)
C22	0.0143 (14)	0.0203 (16)	0.0168 (15)	0.0014 (12)	0.0060 (12)	0.0011 (12)
C23	0.0264 (17)	0.0196 (17)	0.0171 (16)	−0.0029 (13)	0.0037 (13)	0.0041 (13)
C24	0.0235 (16)	0.0286 (19)	0.0169 (16)	0.0020 (14)	0.0037 (13)	0.0030 (13)
C25	0.0208 (16)	0.0214 (16)	0.0179 (16)	0.0013 (13)	0.0029 (13)	0.0041 (12)
C26	0.0257 (17)	0.0169 (16)	0.0140 (15)	0.0060 (13)	0.0055 (13)	0.0046 (12)
C27	0.0261 (17)	0.0171 (16)	0.0192 (16)	0.0018 (13)	0.0040 (13)	0.0006 (13)
C28	0.0233 (16)	0.0219 (17)	0.0119 (15)	−0.0025 (13)	0.0016 (12)	−0.0030 (12)
C29	0.0204 (15)	0.0205 (17)	0.0084 (14)	0.0026 (13)	0.0032 (11)	0.0009 (11)
C30	0.0224 (16)	0.0180 (16)	0.0053 (13)	0.0006 (12)	0.0049 (11)	0.0006 (11)
C31	0.0182 (14)	0.0286 (17)	0.0109 (14)	−0.0019 (14)	0.0053 (11)	0.0001 (13)
C32	0.0171 (16)	0.0274 (18)	0.0256 (18)	0.0070 (13)	0.0065 (13)	−0.0021 (14)
C33	0.0201 (16)	0.0231 (17)	0.0137 (15)	0.0072 (13)	0.0011 (12)	0.0013 (13)
O1	0.061 (4)	0.047 (4)	0.024 (3)	0.020 (3)	0.011 (3)	0.005 (3)
C34	0.054 (5)	0.037 (4)	0.031 (3)	0.019 (4)	0.007 (3)	0.002 (3)
C35	0.047 (4)	0.040 (4)	0.026 (3)	0.013 (4)	0.010 (3)	0.004 (3)
C36	0.048 (4)	0.053 (5)	0.030 (3)	0.009 (4)	0.004 (3)	−0.004 (3)
C37	0.036 (4)	0.042 (4)	0.030 (3)	0.005 (3)	0.010 (3)	0.003 (3)
O1'	0.055 (4)	0.038 (3)	0.028 (3)	0.015 (3)	0.019 (3)	0.009 (2)
C34'	0.053 (5)	0.039 (4)	0.030 (3)	0.014 (4)	0.006 (4)	0.006 (3)
C35'	0.048 (4)	0.039 (4)	0.026 (3)	0.017 (4)	0.011 (4)	0.001 (3)
C36'	0.046 (4)	0.037 (4)	0.027 (3)	0.005 (4)	0.007 (3)	0.006 (3)
C37'	0.054 (4)	0.054 (4)	0.032 (3)	0.016 (4)	0.010 (4)	−0.005 (3)

### Geometric parameters (Å, º)

**Table:**

Ni01—C1	1.909 (3)	C22—C25	1.500 (4)
Ni01—C4	1.916 (3)	C23—H23A	0.9800
Ni01—C30	2.045 (3)	C23—H23B	0.9800
Ni01—C29	2.051 (3)	C23—H23C	0.9800
Ni01—C26	2.123 (3)	C24—H24A	0.9800
Ni01—C33	2.145 (3)	C24—H24B	0.9800
N1—C1	1.377 (4)	C24—H24C	0.9800
N1—C2	1.390 (4)	C25—H25A	0.9800
N1—C8	1.437 (4)	C25—H25B	0.9800
N2—C1	1.374 (3)	C25—H25C	0.9800
N2—C3	1.384 (4)	C26—C33	1.374 (4)
N2—C7	1.451 (4)	C26—C27	1.510 (4)
N3—C4	1.379 (3)	C26—H26A	0.951 (18)
N3—C5	1.388 (3)	C27—C28	1.536 (4)
N3—C7	1.444 (3)	C27—H27A	0.9900
N4—C4	1.374 (3)	C27—H27B	0.9900
N4—C6	1.395 (3)	C28—C29	1.523 (4)
N4—C17	1.441 (3)	C28—H28A	0.9900
C2—C3	1.344 (4)	C28—H28B	0.9900
C2—H2	0.9500	C29—C30	1.411 (4)
C3—H3	0.9500	C29—H29A	0.960 (18)
C5—C6	1.341 (4)	C30—C31	1.512 (4)
C5—H5	0.9500	C30—H30A	0.956 (19)
C6—H6	0.9500	C31—C32	1.539 (4)
C7—H7A	0.9900	C31—H31A	0.9900
C7—H7B	0.9900	C31—H31B	0.9900
C8—C13	1.392 (4)	C32—C33	1.516 (4)
C8—C9	1.394 (4)	C32—H32A	0.9900
C9—C10	1.397 (4)	C32—H32B	0.9900
C9—C14	1.503 (4)	C33—H33A	0.960 (19)
C10—C11	1.379 (4)	O1—C34	1.415 (11)
C10—H10	0.9500	O1—C37	1.417 (8)
C11—C12	1.392 (4)	C34—C35	1.506 (11)
C11—C15	1.510 (4)	C34—H34A	0.9900
C12—C13	1.387 (4)	C34—H34B	0.9900
C12—H12	0.9500	C35—C36	1.503 (12)
C13—C16	1.505 (4)	C35—H35A	0.9900
C14—H14A	0.9800	C35—H35B	0.9900
C14—H14B	0.9800	C36—C37	1.512 (11)
C14—H14C	0.9800	C36—H36A	0.9900
C15—H15A	0.9800	C36—H36B	0.9900
C15—H15B	0.9800	C37—H37A	0.9900
C15—H15C	0.9800	C37—H37B	0.9900
C16—H16A	0.9800	O1'—C37'	1.344 (9)
C16—H16B	0.9800	O1'—C34'	1.420 (10)
C16—H16C	0.9800	C34'—C35'	1.524 (11)
C17—C18	1.389 (4)	C34'—H34C	0.9900
C17—C22	1.400 (4)	C34'—H34D	0.9900
C18—C19	1.390 (4)	C35'—C36'	1.526 (11)
C18—C23	1.512 (4)	C35'—H35C	0.9900
C19—C20	1.390 (4)	C35'—H35D	0.9900
C19—H19	0.9500	C36'—C37'	1.514 (11)
C20—C21	1.385 (4)	C36'—H36C	0.9900
C20—C24	1.512 (4)	C36'—H36D	0.9900
C21—C22	1.394 (4)	C37'—H37C	0.9900
C21—H21	0.9500	C37'—H37D	0.9900
			
C1—Ni01—C4	91.51 (12)	C20—C24—H24A	109.5
C1—Ni01—C30	107.31 (12)	C20—C24—H24B	109.5
C4—Ni01—C30	142.09 (12)	H24A—C24—H24B	109.5
C1—Ni01—C29	143.24 (12)	C20—C24—H24C	109.5
C4—Ni01—C29	107.85 (12)	H24A—C24—H24C	109.5
C30—Ni01—C29	40.29 (11)	H24B—C24—H24C	109.5
C1—Ni01—C26	125.47 (12)	C22—C25—H25A	109.5
C4—Ni01—C26	93.00 (12)	C22—C25—H25B	109.5
C30—Ni01—C26	101.53 (12)	H25A—C25—H25B	109.5
C29—Ni01—C26	85.37 (12)	C22—C25—H25C	109.5
C1—Ni01—C33	100.31 (12)	H25A—C25—H25C	109.5
C4—Ni01—C33	124.47 (12)	H25B—C25—H25C	109.5
C30—Ni01—C33	84.99 (12)	C33—C26—C27	125.9 (3)
C29—Ni01—C33	94.06 (12)	C33—C26—Ni01	72.11 (18)
C26—Ni01—C33	37.55 (11)	C27—C26—Ni01	106.80 (19)
C1—N1—C2	112.4 (2)	C33—C26—H26A	116.6 (17)
C1—N1—C8	125.1 (2)	C27—C26—H26A	115.6 (17)
C2—N1—C8	122.3 (2)	Ni01—C26—H26A	105.1 (17)
C1—N2—C3	113.4 (2)	C26—C27—C28	113.8 (3)
C1—N2—C7	120.2 (2)	C26—C27—H27A	108.8
C3—N2—C7	126.4 (2)	C28—C27—H27A	108.8
C4—N3—C5	112.8 (2)	C26—C27—H27B	108.8
C4—N3—C7	121.0 (2)	C28—C27—H27B	108.8
C5—N3—C7	126.1 (2)	H27A—C27—H27B	107.7
C4—N4—C6	112.1 (2)	C29—C28—C27	112.3 (2)
C4—N4—C17	126.2 (2)	C29—C28—H28A	109.1
C6—N4—C17	121.7 (2)	C27—C28—H28A	109.1
N2—C1—N1	101.4 (2)	C29—C28—H28B	109.1
N2—C1—Ni01	119.8 (2)	C27—C28—H28B	109.1
N1—C1—Ni01	138.6 (2)	H28A—C28—H28B	107.9
C3—C2—N1	106.9 (3)	C30—C29—C28	122.4 (3)
C3—C2—H2	126.5	C30—C29—Ni01	69.64 (16)
N1—C2—H2	126.5	C28—C29—Ni01	111.99 (19)
C2—C3—N2	105.9 (3)	C30—C29—H29A	119.2 (17)
C2—C3—H3	127.0	C28—C29—H29A	113.7 (17)
N2—C3—H3	127.0	Ni01—C29—H29A	109.8 (17)
N4—C4—N3	101.8 (2)	C29—C30—C31	123.1 (3)
N4—C4—Ni01	139.2 (2)	C29—C30—Ni01	70.07 (16)
N3—C4—Ni01	119.02 (19)	C31—C30—Ni01	111.30 (19)
C6—C5—N3	106.2 (2)	C29—C30—H30A	116.0 (19)
C6—C5—H5	126.9	C31—C30—H30A	116.3 (19)
N3—C5—H5	126.9	Ni01—C30—H30A	108.9 (19)
C5—C6—N4	107.1 (3)	C30—C31—C32	113.9 (2)
C5—C6—H6	126.5	C30—C31—H31A	108.8
N4—C6—H6	126.5	C32—C31—H31A	108.8
N3—C7—N2	110.2 (2)	C30—C31—H31B	108.8
N3—C7—H7A	109.6	C32—C31—H31B	108.8
N2—C7—H7A	109.6	H31A—C31—H31B	107.7
N3—C7—H7B	109.6	C33—C32—C31	114.1 (2)
N2—C7—H7B	109.6	C33—C32—H32A	108.7
H7A—C7—H7B	108.1	C31—C32—H32A	108.7
C13—C8—C9	121.6 (3)	C33—C32—H32B	108.7
C13—C8—N1	117.8 (3)	C31—C32—H32B	108.7
C9—C8—N1	120.5 (3)	H32A—C32—H32B	107.6
C8—C9—C10	117.5 (3)	C26—C33—C32	123.8 (3)
C8—C9—C14	121.9 (3)	C26—C33—Ni01	70.34 (17)
C10—C9—C14	120.6 (3)	C32—C33—Ni01	109.86 (19)
C11—C10—C9	122.4 (3)	C26—C33—H33A	118.2 (17)
C11—C10—H10	118.8	C32—C33—H33A	115.3 (17)
C9—C10—H10	118.8	Ni01—C33—H33A	106.4 (17)
C10—C11—C12	118.3 (3)	C34—O1—C37	110.5 (7)
C10—C11—C15	122.1 (3)	O1—C34—C35	107.6 (9)
C12—C11—C15	119.6 (3)	O1—C34—H34A	110.2
C13—C12—C11	121.5 (3)	C35—C34—H34A	110.2
C13—C12—H12	119.3	O1—C34—H34B	110.2
C11—C12—H12	119.3	C35—C34—H34B	110.2
C12—C13—C8	118.6 (3)	H34A—C34—H34B	108.5
C12—C13—C16	120.4 (3)	C36—C35—C34	103.4 (8)
C8—C13—C16	120.9 (3)	C36—C35—H35A	111.1
C9—C14—H14A	109.5	C34—C35—H35A	111.1
C9—C14—H14B	109.5	C36—C35—H35B	111.1
H14A—C14—H14B	109.5	C34—C35—H35B	111.1
C9—C14—H14C	109.5	H35A—C35—H35B	109.1
H14A—C14—H14C	109.5	C35—C36—C37	103.9 (9)
H14B—C14—H14C	109.5	C35—C36—H36A	111.0
C11—C15—H15A	109.5	C37—C36—H36A	111.0
C11—C15—H15B	109.5	C35—C36—H36B	111.0
H15A—C15—H15B	109.5	C37—C36—H36B	111.0
C11—C15—H15C	109.5	H36A—C36—H36B	109.0
H15A—C15—H15C	109.5	O1—C37—C36	106.3 (7)
H15B—C15—H15C	109.5	O1—C37—H37A	110.5
C13—C16—H16A	109.5	C36—C37—H37A	110.5
C13—C16—H16B	109.5	O1—C37—H37B	110.5
H16A—C16—H16B	109.5	C36—C37—H37B	110.5
C13—C16—H16C	109.5	H37A—C37—H37B	108.7
H16A—C16—H16C	109.5	C37'—O1'—C34'	110.2 (7)
H16B—C16—H16C	109.5	O1'—C34'—C35'	105.3 (8)
C18—C17—C22	121.9 (3)	O1'—C34'—H34C	110.7
C18—C17—N4	119.4 (2)	C35'—C34'—H34C	110.7
C22—C17—N4	118.6 (3)	O1'—C34'—H34D	110.7
C17—C18—C19	118.2 (3)	C35'—C34'—H34D	110.7
C17—C18—C23	122.2 (3)	H34C—C34'—H34D	108.8
C19—C18—C23	119.6 (3)	C34'—C35'—C36'	102.8 (9)
C18—C19—C20	121.8 (3)	C34'—C35'—H35C	111.2
C18—C19—H19	119.1	C36'—C35'—H35C	111.2
C20—C19—H19	119.1	C34'—C35'—H35D	111.2
C21—C20—C19	118.4 (3)	C36'—C35'—H35D	111.2
C21—C20—C24	120.7 (3)	H35C—C35'—H35D	109.1
C19—C20—C24	120.8 (3)	C37'—C36'—C35'	100.3 (8)
C20—C21—C22	122.0 (3)	C37'—C36'—H36C	111.7
C20—C21—H21	119.0	C35'—C36'—H36C	111.7
C22—C21—H21	119.0	C37'—C36'—H36D	111.7
C21—C22—C17	117.6 (3)	C35'—C36'—H36D	111.7
C21—C22—C25	120.8 (3)	H36C—C36'—H36D	109.5
C17—C22—C25	121.5 (3)	O1'—C37'—C36'	111.1 (7)
C18—C23—H23A	109.5	O1'—C37'—H37C	109.4
C18—C23—H23B	109.5	C36'—C37'—H37C	109.4
H23A—C23—H23B	109.5	O1'—C37'—H37D	109.4
C18—C23—H23C	109.5	C36'—C37'—H37D	109.4
H23A—C23—H23C	109.5	H37C—C37'—H37D	108.0
H23B—C23—H23C	109.5		
			
C3—N2—C1—N1	−0.4 (3)	C9—C8—C13—C16	−177.7 (3)
C7—N2—C1—N1	−179.3 (2)	N1—C8—C13—C16	4.4 (4)
C3—N2—C1—Ni01	−175.9 (2)	C4—N4—C17—C18	115.5 (3)
C7—N2—C1—Ni01	5.2 (3)	C6—N4—C17—C18	−67.4 (4)
C2—N1—C1—N2	0.8 (3)	C4—N4—C17—C22	−67.7 (4)
C8—N1—C1—N2	175.4 (3)	C6—N4—C17—C22	109.3 (3)
C2—N1—C1—Ni01	174.8 (3)	C22—C17—C18—C19	0.2 (4)
C8—N1—C1—Ni01	−10.5 (5)	N4—C17—C18—C19	176.8 (2)
C1—N1—C2—C3	−0.8 (3)	C22—C17—C18—C23	−177.6 (3)
C8—N1—C2—C3	−175.7 (3)	N4—C17—C18—C23	−0.9 (4)
N1—C2—C3—N2	0.5 (3)	C17—C18—C19—C20	−0.4 (4)
C1—N2—C3—C2	−0.1 (3)	C23—C18—C19—C20	177.4 (3)
C7—N2—C3—C2	178.8 (3)	C18—C19—C20—C21	0.8 (4)
C6—N4—C4—N3	−0.8 (3)	C18—C19—C20—C24	−178.3 (3)
C17—N4—C4—N3	176.5 (2)	C19—C20—C21—C22	−1.1 (4)
C6—N4—C4—Ni01	178.4 (3)	C24—C20—C21—C22	178.0 (3)
C17—N4—C4—Ni01	−4.3 (5)	C20—C21—C22—C17	0.9 (4)
C5—N3—C4—N4	0.6 (3)	C20—C21—C22—C25	−179.5 (3)
C7—N3—C4—N4	178.6 (2)	C18—C17—C22—C21	−0.4 (4)
C5—N3—C4—Ni01	−178.8 (2)	N4—C17—C22—C21	−177.1 (2)
C7—N3—C4—Ni01	−0.8 (4)	C18—C17—C22—C25	−180.0 (3)
C4—N3—C5—C6	−0.2 (3)	N4—C17—C22—C25	3.4 (4)
C7—N3—C5—C6	−178.1 (3)	C33—C26—C27—C28	−46.3 (4)
N3—C5—C6—N4	−0.3 (3)	Ni01—C26—C27—C28	33.5 (3)
C4—N4—C6—C5	0.7 (3)	C26—C27—C28—C29	−32.1 (4)
C17—N4—C6—C5	−176.7 (3)	C27—C28—C29—C30	92.8 (3)
C4—N3—C7—N2	53.4 (3)	C27—C28—C29—Ni01	13.6 (3)
C5—N3—C7—N2	−128.9 (3)	C28—C29—C30—C31	−0.7 (4)
C1—N2—C7—N3	−56.0 (3)	Ni01—C29—C30—C31	103.0 (3)
C3—N2—C7—N3	125.3 (3)	C28—C29—C30—Ni01	−103.7 (3)
C1—N1—C8—C13	−91.4 (3)	C29—C30—C31—C32	−52.8 (4)
C2—N1—C8—C13	82.8 (4)	Ni01—C30—C31—C32	26.7 (3)
C1—N1—C8—C9	90.7 (4)	C30—C31—C32—C33	−24.3 (4)
C2—N1—C8—C9	−95.1 (3)	C27—C26—C33—C32	−3.1 (5)
C13—C8—C9—C10	0.7 (4)	Ni01—C26—C33—C32	−101.2 (3)
N1—C8—C9—C10	178.6 (3)	C27—C26—C33—Ni01	98.1 (3)
C13—C8—C9—C14	−178.9 (3)	C31—C32—C33—C26	89.1 (4)
N1—C8—C9—C14	−1.1 (4)	C31—C32—C33—Ni01	10.0 (3)
C8—C9—C10—C11	−1.8 (4)	C37—O1—C34—C35	6.5 (17)
C14—C9—C10—C11	177.9 (3)	O1—C34—C35—C36	−22.2 (19)
C9—C10—C11—C12	1.4 (4)	C34—C35—C36—C37	28.4 (18)
C9—C10—C11—C15	−178.1 (3)	C34—O1—C37—C36	12.0 (16)
C10—C11—C12—C13	0.1 (4)	C35—C36—C37—O1	−25.4 (16)
C15—C11—C12—C13	179.6 (3)	C37'—O1'—C34'—C35'	−17.7 (17)
C11—C12—C13—C8	−1.1 (4)	O1'—C34'—C35'—C36'	30.1 (18)
C11—C12—C13—C16	177.3 (3)	C34'—C35'—C36'—C37'	−29.8 (17)
C9—C8—C13—C12	0.7 (4)	C34'—O1'—C37'—C36'	−2.5 (16)
N1—C8—C13—C12	−177.2 (3)	C35'—C36'—C37'—O1'	21.2 (17)

## References

[bb1] Arduengo, A. J. (1999). *Acc. Chem. Res.* **32**, 913–921.

[bb2] Brendel, M., Braun, C., Rominger, F. & Hofmann, P. (2014). *Angew. Chem. Int. Ed.* **53**, 8741–8745. 10.1002/anie.201401024 24849867

[bb3] Bruker (2017). *APEX3* and *SAINT*. Bruker AXS Inc., Madison, Wisconsin, USA.

[bb4] Douthwaite, R. E., Haüssinger, D., Green, M. L. H., Silcock, P. J., Gomes, P. T., Martins, A. M. & Danopoulos, A. A. (1999). *Organometallics*, **18**, 4584–4590.

[bb5] Gardiner, M. G., Herrmann, W. A., Reisinger, C., Schwarz, J. & Spiegler, M. (1999). *J. Organomet. Chem.* **572**, 239–247.

[bb6] Groom, C. R., Bruno, I. J., Lightfoot, M. P. & Ward, S. C. (2016). *Acta Cryst.* B**72**, 171–179. 10.1107/S2052520616003954 PMC4822653 27048719

[bb7] Harrold, N. D. & Hillhouse, G. L. (2013). *Chem. Sci.* **4**, 4011–4015.

[bb8] Herrmann, W. A., Schwarz, J., Gardiner, M. G. & Spiegler, M. (1999). *J. Organomet. Chem.* **575**, 80–86.

[bb9] Hopkinson, M. N., Richter, C., Schedler, M. & Glorius, F. (2014). *Nature*, **510**, 485–496. 10.1038/nature13384 24965649

[bb10] Huffer, A., Jeffery, B., Waller, B. J. & Danopoulos, A. A. (2013). *C. R. Chim.* **16**, 557–565.

[bb11] Krause, L., Herbst-Irmer, R., Sheldrick, G. M. & Stalke, D. (2015). *J. Appl. Cryst.* **48**, 3–10. 10.1107/S1600576714022985 PMC4453166 26089746

[bb12] Liu, J., Chen, J., Zhao, J., Zhao, Y., Li, L. & Zhang, H. (2003). *Synthesis*, pp. 2661–2666.

[bb13] Lummiss, J. A. M., Higman, C. S., Fyson, D. L., McDonald, R. & Fogg, D. E. (2015). *Chem. Sci.* **6**, 6739–6746. 10.1039/c5sc02592c PMC5947514 29861923

[bb14] Macrae, C. F., Edgington, P. R., McCabe, P., Pidcock, E., Shields, G. P., Taylor, R., Towler, M. & van de Streek, J. (2006). *J. Appl. Cryst.* **39**, 453–457.

[bb15] SciFinder (2018). Chemical Abstracts Service: Colombus, OH, 2010; RN 58-08-2 (accessed August 10, 2018).

[bb16] Sheldrick, G. M. (2015*a*). *Acta Cryst.* A**71**, 3–8.

[bb17] Sheldrick, G. M. (2015*b*). *Acta Cryst.* C**71**, 3–8.

[bb18] Westrip, S. P. (2010). *J. Appl. Cryst.* **43**, 920–925.

